# Microwave induced synthesis of ZnO nanorods and their efficacy as a drug carrier with profound anticancer and antibacterial properties

**DOI:** 10.1016/j.toxrep.2019.01.006

**Published:** 2019-01-29

**Authors:** Pritam Sadhukhan, Mousumi Kundu, Shallu Rana, Raj Kumar, Joydeep Das, Parames C. Sil

**Affiliations:** a Division of Molecular Medicine, Bose Institute, P-1/12, CIT Scheme VII M, Kolkata 700054, India; b Shoolini University of Biotechnology and Management Sciences, Bajhol, PO Sultanpur, Distt. Solan- 173229 (HP), India

**Keywords:** Anticancer, Antibacterial, Drug delivery, Quercetin, Zinc oxide nanorods

## Abstract

In the present study, we report the microwave-induced synthesis of fluorescent zinc oxide nanorods (ZnO) and their usage as a cargo material to carry hydrophobic drug, quercetin. TEM and SEM showed the rod-shape morphology of our synthesized ZnO. XRD showed several diffraction peaks correspond to a hexagonal wurtzite structure. The optical and chemical natures of these nanorods were also confirmed from the UV-vis (showed a distinct absorption bands from 361 to 395 nm) and FTIR spectrum (showed absorption band specific to Zn—O stretching). The synthesized ZnO also showed fluorescence emission at around 550 nm when excited under UV irradiation. Quercetin was loaded onto ZnO surface via employing a metal ion-ligand coordination bond, (ZnO/QR), which exhibit pH-sensitive release behavior. ZnO/QR displayed superior drug loading content (42%) and loading efficiency (72.4%). *in vitro* assays showed that ZnO/QR exhibited higher anticancer, as well as antibacterial activities compared with free quercetin and ZnO. All these results highlight the synthesis of ZnO nanorods under microwave irradiation, which can be used as a plausible therapeutic option for bioimaging and drug delivery purpose.

## Highlights

• Novel ZnO nanorods were synthesized and loaded with hydrophobic biomolecule quercetin.
• The synthesized nanohybrids showed pH sensitive drug release property.
• The nanohybrids have intrinsic fluorescent properties, beneficial for bioimaging.
• The nanohybrids exhibited potential anticancer and antimicrobial activities.

## Graphical abstract

**Figure:**
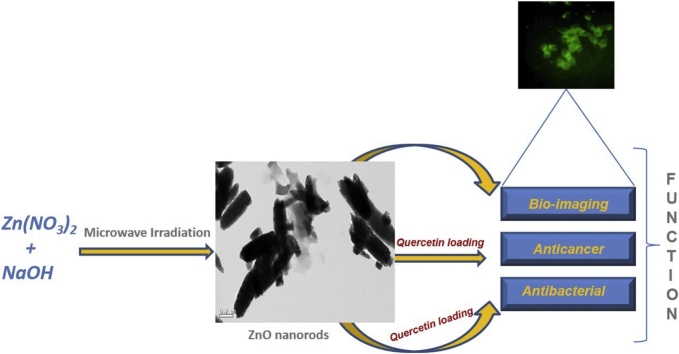

## 1 Introduction

In recent years, the advancement of nanotechnology via designing of functional nanomaterials with novel properties for potential applications in chemical, biological, and technological domains have attracted much attention. Nanocrystalline zinc oxide (ZnO) is a n-type semiconductor having relatively large band gap around 3.37 eV along with high excitation energy of binding (approximately 60 meV) at room temperature [1]. Owing to these noteworthy properties, ZnO is extensively used in several areas, such as thin film transistors [[2], [3], [4]], piezoelectric devices [5,6], UV/ozone sensor [[7], [8], [9]], dye-sensitized solar cells [10,11] and glucose sensor [12]. However, the chemical and physical properties of ZnO nanoparticles are closely dependent on their shape, size, morphology and crystallinity of the synthesized nanostructures [13].

ZnO nanoparticles show brilliant photocatalytic characteristic to decrease contaminants in the environment. The basic principle of photocatalysis involves the promotion of electron (e^−^) from valence band to conduction band and creation of hole (h^+^) under solar light irradiation, (ii) migration of the (e^−^/h^+^) pair to the ZnO surface, (iii) generation of hydroxyl radical (^•^OH) via redox reaction, and decomposition of the pollutant by highly oxidizing ·OH [14,15]. However, ZnO is considered more efficient photocatalyst than TiO_2_ under visible light irradiation because of its lower cost of production, nontoxicity and higher quantum efficiency [14,16]. Therefore, ZnO are emerged as a promising alternative for waste water treatment and disinfection.

ZnO nanorods also behave as anticancer, antibacterial or antifungal agents [[17], [18], [19]]. A number of studies demonstrated that the ZnO induce significant cytotoxicity, apoptosis, and autophagy in several human cancer cells via reactive oxygen species (ROS) generation and preferential dissolution of Zn^2+^ ions in mild acidic cancer microenvironment, while sparing normal cells due to pH difference [17,20,21]. The antibacterial properties of ZnO are attributed to its electrostatic interaction between the nanoparticles and cell surface/membrane of the bacterium [18]. Nanoparticles penetrate the cell wall of microbes by using the ion channels and carrier proteins, can able to bind with different intracellular organelles and thus affect the metabolic processes through the formation and accumulation of ROS. The fungal and bacterial lipid bilayer gets disrupted due to the association of ZnO and cell membrane resulting in the leakage of the cytoplasmic contents [22]. However, the size and shape of the nanoparticles again has a critical role in conferring its cytotoxicity.

ZnO have significant achievements in therapeutic applications, such as bioimaging and drug carrier. Recently, zinc oxide-based nanostructures have gained much attention in the pre-clinical research of drug delivery systems (DDS). Different research reports suggested ZnO as safe by the FDA, USA. However, the successful therapeutic application of ZnO in DDS is still at a preliminary stage. There are only few reports of using acid-degradable ZnO nanoparticles for successful delivery of doxorubicin into cancer cells [20,[23], [24], [25], [26]]. Besides, several other researchers also demonstrated the anti-cancer activity of ZnO nanoparticles, loaded with isotretinoin [27], curcumin [[28], [29], [30]] and paclitaxel [31] respectively. Quercetin is a naturally occurring bioactive compound with significant antioxidant, anticancer activity and can modulate pro-survival signaling cascades in a cell specific manner. Besides, it has several other pharmacological effects including cardioprotective, bacteriostatic, and antiviral activities [[32], [33], [34], [35]]. However, quercetin has limitation in its usage mainly due to poor bioavailability and high metabolic activity [[35], [36], [37], [38]]. Hence, quercetin should be modified with some other agents to overcome those shortcomings. There is no previous report regarding the use of quercetin-loaded ZnO nanorods as an efficient anticancer and antibacterial agent.

In the current study, ZnO nanorods were synthesized under microwave induced irradiation, and the structural and optical characterizations of the synthesized nanorods were performed by transmission electron microscopy (TEM) [8], scanning electron microscopy (SEM), energy dispersive X-Rayspectroscopy (EDS), X-Ray diffraction (XRD), Fourier transform infrared (FTIR), UV–vis and fluorescence spectroscopy [19]. Finally, the ZnO nanorods were conjugated with the well-known hydrophobic anticancer and antibacterial agent quercetin (QR) and investigated the anticancer as well as antibacterial potential of ZnO/QR nanorods in attenuating proliferation of breast cancer cells and growth inhibition of *E.coli* bacteria *in vitro*. Herein, we hypothesize that a significant anticancer and antibacterial potency can be achieved by combining the QR and the anti-tumorigenic/antibacterial property of ZnO to synthesis a hybrid (ZnO/QR).

## 2 Materials and methods

### 2.1 Materials

Zinc nitrate was purchased from Qualikens, India. Sodium hydroxide (NaOH) was purchased from Ranken, India. Dimethyl sulfoxide (DMSO) and sodium borohydride were purchased from LOBA Chemie, India. Quercetin was purchased from Himedia, India. All the chemicals used were of analytical grade and used without any further purification.

### 2.2 Synthesis of ZnO nanorods

ZnO nanorods were synthesized by using zinc nitrate (0.6 g) and NaOH (0.16 g) mixture in 100 ml water under stirring condition for 30 min. The pH of the solution was maintained at 8.3. The mixture of zinc nitrate and Sodium hydroxide solution was then shifted to a domestic micro-oven for heating at different time points (from 1 to 5 min.). The nanoparticles were named as ZnO1, ZnO2, ZnO3, ZnO4, and ZnO5 according to the time of heating. After completion of microwave heating, the white product was cooled at room temperature and centrifuged at 4000 rpm for 10 min. The product was further washed with ethanol for the removal of impurities and dried at 70 °C in hot air oven.

### 2.3 Synthesis of quercetin loaded ZnO nanorods

Quercetin loaded ZnO nanorods (ZnO/QR) were synthesized by mixing ZnO nanorods (100 mg) and quercetin (100 mg) in 10 mL DMSO. For drug loading experiments, only ZnO4 was used. The solution mixture was first sonicated for 10 min. and allowed to stir over night. After the required time point, the yellow product was centrifuged at 4000 rpm for 10 min. The product was further washed with DMSO to remove the unbound quercetin and air dried. This process yielded a fine powder of quercetin loaded ZnO nanorods (ZnO/QR). The drug loading efficiency (DLE) and drug-loading content (DLC) were quantified by UV-VIS spectroscopy [26] and thermo gravimetric analysis (TGA) with a heating rate of 10 °C/min in N_2_ atmosphere.

### 2.4 Characterization of synthesized nanorods

The primary sizes of ZnO and ZnO/QR nanorods were measured by TEM and SEM. The synthesized ZnO was characterized by EDS, FTIR and XRD analysis. The UV-VIS and fluorescence spectra of the nanorods were acquired using an UV–vis spectrophotometer and fluorimeter. The zeta potentials and hydrodynamic sizes of the nanorods were obtained using the Zeta-sizer instrument. The fluorescent nature of the ZnO nanorods were also verified by fluorescent microscope (Fluorescent microscope, Nikon).

### 2.5 Cell culture

The MCF-7 cells (human breast adenocarcinoma) were used in subsequent experiments to evaluate the uptake efficiency and the anticancer efficacy of the synthesized nanoparticles. The cell line was obtained from NCCS, India and cultured using the standard RPMI media with 10% fetal bovine serum in a humidified incubator at 37 ^о^C.

### 2.6 Cellular uptake of ZnO nanorods

The cellular uptake efficiency of ZnO nanoparticles in the MCF-7 cells were investigated using fluorescence microscopy. Briefly, the 10^5^ cells per well were plated on a poly l-lysine coated coverslip placed in 35 mm petri dish and incubated overnight at normal conditions. The cells were then exposed with 11.6 μg/mL ZnO nanoparticles for 6 h. Followed by this, the cells were washed 1X PBS and fixed with freshly prepared paraformaldehyde (4%). The cells were then stained with DAPI. Finally, the coverslips were mounted on a glass slides and the uptake of the fluorescent nanorods was observed using 40X magnification.

### 2.7 *In vitro* quercetin release from ZnO/QR nanorods quercetin release from ZnO/QR nanorods

The *in vitro* release of drug candidate from ZnO/QR nanorods were investigated in neutral and acidic buffer using a dialysis diffusion technique. To quantify the amount of quercetin release, ZnO/QR nanorods (equivalent quercetin concentration of 1 mg/mL) was resuspended in 10 mL PBS, taken in a dialysis bags (MWCO 3500 Da), and incubated at 37 °C in PBS with shaking at 100 rpm. Thereafter, a portion of the incubated solution was taken out after fixed time interval and replaced with fresh PBS. The drug release was determined by measuring the absorbance at wavelength of 375 nm.

### 2.8 Cytotoxicity assay

Cells were plated into a 96-well plates at a density of 1.5 × 10^4^ cells per well and incubated overnight at normal conditions. The cells were then exposed to different concentrations of ZnO (2.9–29 μg/ml), QR (2.1–21 μg/mL), or ZnO/QR (5–50 μg/mL) for 48 h. The cell viability assay was performed using MTT assay following the protocol described elsewhere. Briefly, after the nanoparticle exposure the cells were incubated in the MTT solution (0.5 mg/ml) for 4 h. Followed by this, 100 μl of DMSO was added to each well to dissolve the formazan crystals. Finally, the absorbance was recorded at 570 nm. The % of cell viability was expressed with a comparison to the control cells [39]. Phase contrast micrographs of the nanoparticles exposed cells were also compared to control cells.

### 2.9 Assay for intracellular reactive oxygen species (ROS) and mitochondrial membrane potential (MMP)

MCF-7 Cells were seeded in a flat bottom 35 mm culture dish and incubated overnight at normal conditions. The cells were then exposed to ZnO, QR or ZnO/QR separately for 48 h. For the estimation of intracellular ROS and MMP the nanoparticles exposed cells were stained with H_2_-DCFDA (due to formation of DCF) and Rhodamine 123 (due to accumulation in healthy mitochondria) respectively following the protocol described elsewhere. After an incubation period of 30 min., the cells were analyzed in BD-FACS instrument (by the change in green fluorescence) and the data were analyzed by using the FACS suite software [39].

### 2.10 Antibacterial assay

Dose and time dependent growth inhibition activity of ZnO/QR was carried out on gram negative *E.coli* bacteria. The assay was performed according to the following protocol. Briefly, from an overnight grown culture, 10 μl of inoculums was added to a fresh culture medium supplemented with different concentrations of either ZnO (0–72 μg/ml), QR (0–48 μg/ml) or ZnO/QR (0–120 μg/ml). LB with only particles was considered as blank and LB with only inoculum is considered as controls in the experiments. All the tubes were incubated in a shaker at 37 °C. Finally, the growth of the bacteria was measured using a spectrophotometer at 600 nm.

### 2.11 Adherence of nanoparticles on bacterial cells

Bacterial cells growing at log phase were incubated with ZnO/QR at a dose of 80 μg/ml for 6 h. The cells were then analyzed using the FSC and SSC of a BD-FACS instrument. The cell population in the FSC region corresponds to the size of the cells and SSC indicate the cellular granularity.

### 2.12 Statistical analysis

The experiments were performed 3 times, and statistical analyses were carried using ANOVA followed by Tukey test. P < 0.05was considered to be significant.

## 3 Results and discussion

### 3.1 Preparation and characterization of zinc oxide nanorods

**Fig. 1:**
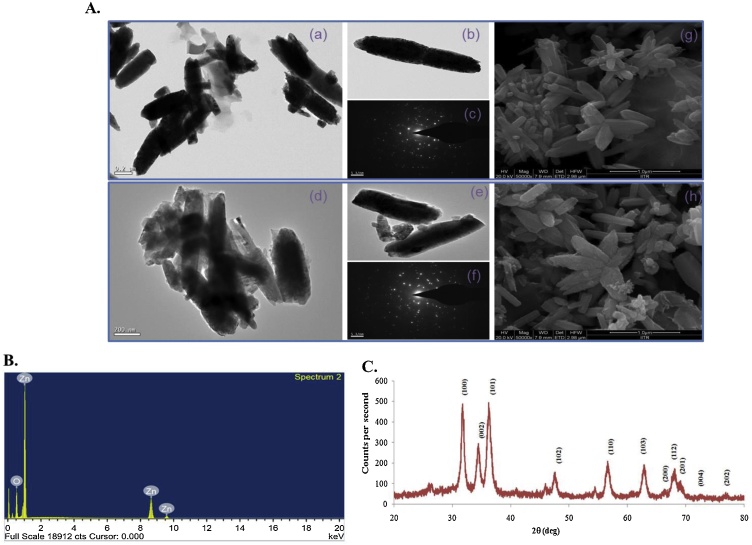
TEM/SEM images, EDS analysis and XRD Pattern of ZnO (ZnO4) and/or ZnO/QR nanorods: (A) (a) and (b) are TEM images of ZnO nanorods. (d) and (e) are TEM images of ZnO/QR nanorods. (c) and (f) are SAED pattern of ZnO and ZnO/QR nanorods. (g) and (h) are SEM images of ZnO and ZnO/QR nanorods. (B) EDS of ZnO4 (ZnO) nanorods. (C) XRD pattern of ZnO4 (ZnO) nanorods.

**Fig. 2:**
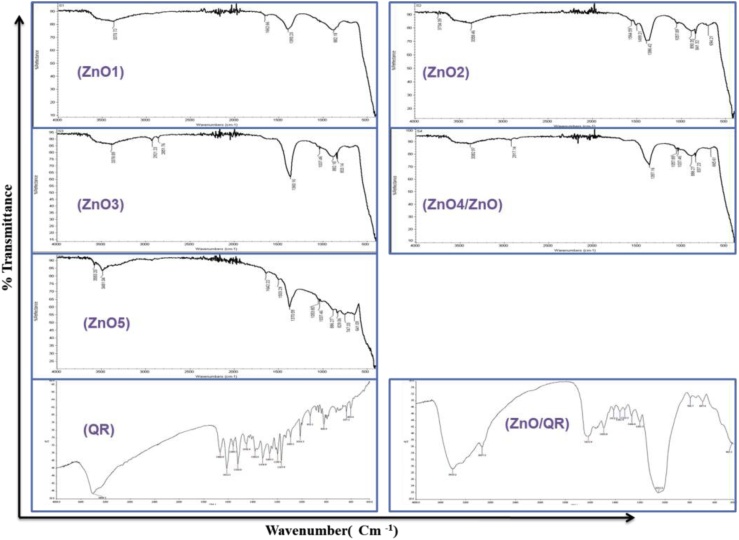
FTIR Spectrum of ZnO nanorods. FTIR spectra of ZnO1, ZnO2, ZnO3, ZnO4(ZnO), ZnO5, ZnO/QR nanorods and quercetin (QR).

Zinc oxide nanorods (ZnO) were synthesized from zinc nitrate considering a hydrothermal growth that has been assisted via microwave irradiation. In our current study we used a water soluble precursor (zinc nitrate) of ZnO instead of frequently used zinc acetate which is alcohol soluble. Therefore, no toxic solvent was used. Besides, we employed short-time microwave heating and avoiding long time refluxing of the reaction mixture as in classical thermal methods. TEM and SEM have been used to investigate the morphology of the newly synthesized ZnO. These analyses indicated mostly the rod like morphologies of the particles, which showed some sort of aggregation (Fig. 1A). Besides, it was also observed that the rod like ZnO nanoparticles have the average diameter in between 100 and 200 nm. However, ZnO flower structures also appeared as evident from the SEM images. The heating time also regulates the morphological or structural changes of the synthesized ZnO nanorods. With increasing the duration of microwave heating, ZnO nanorods are spontaneously changed into nanoflower shape as evident form Pimentel et al [40]. The selected area electrondiffraction (SAED) pattern shows a set of points obtained by diffraction of electrons from various planes of ZnO nanorods, thereby confirming its crystalline nature (Fig. 1A). The chemical property of ZnO was verified from the FTIR spectrum, which showed a characteristic strong absorption at 460 to 470 cm^−1^ due to the Zn—O stretching vibration [20] (Fig. 2). Weak infrared absorption bands were also observed at 3400–3500 cm^−1^ (stretching band) and 1360–1390 cm^−1^ (bend band) due to water molecules adsorbed on the nanoparticle surface [26] (Fig. 2). The synthesized ZnO were also characterized by EDS. The EDS spectrum showed characteristic peaks of Zn and O and was devoid of any impurity peaks (Fig. 1B). We have also checked the crystal structure (by XRD analysis) of the synthesized ZnO (Fig. 1C). The high intensity peaks were observed at 2θ = 31.73^o^, 34.31^o^, 36.02^o^, 47.58^o^, 56.42^o^, 62.70^o^, 66.41^o^, 67.98^o^, 69.12^o^, 72.5^o^ and 76.98^o^ indexing (100), (002), (101), (102), (110), (103), (200), (112), (201), (004) and (202) diffraction planes of ZnO respectively. These diffraction peaks indicate a hexagonal wurtzite crystal structure surface [26].

**Fig. 3:**
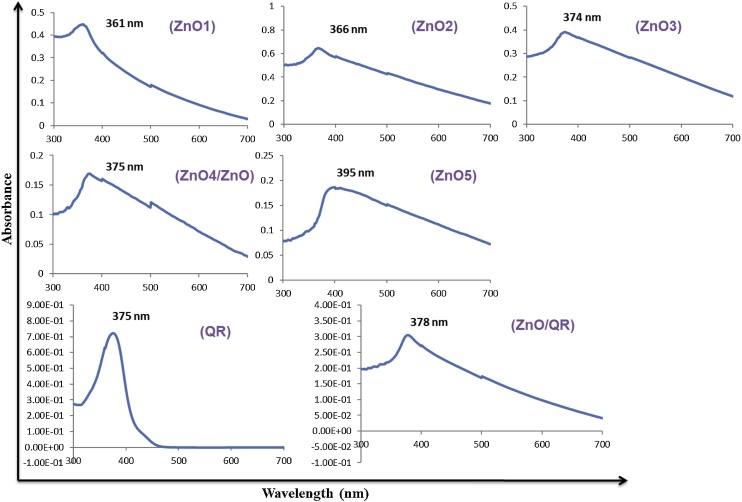
UV-VIS Spectrum of ZnO nanorods. UV-VIS Spectrumof ZnO1, ZnO2, ZnO3, ZnO4(ZnO), ZnO5, ZnO/QR nanorods and quercetin (QR).

**Fig. 4:**
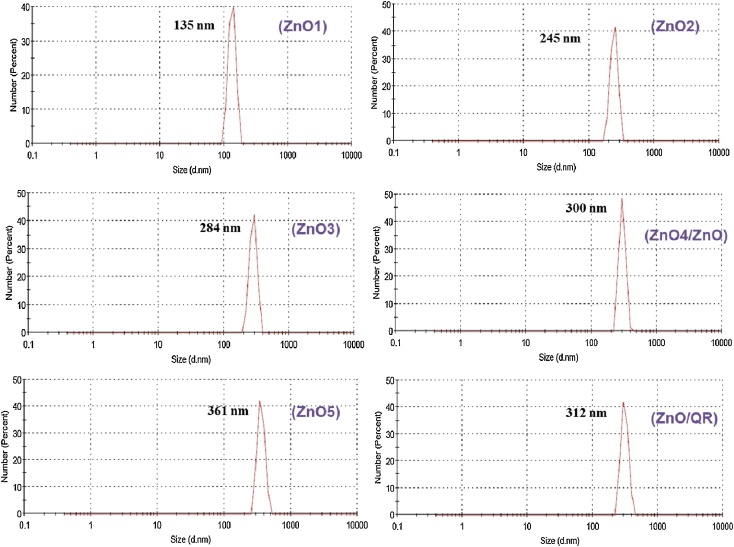
Hydrodynamic size distribution of ZnO nanorods. Hydrodynamic size distribution of ZnO1, ZnO2, ZnO3, ZnO4(ZnO), ZnO5 and ZnO/QR nanorods.

The heating time also regulates the size and optical properties of the synthesized ZnO nanorods. The optical nature of synthesized ZnO were analysed from the UV-VIS spectrum, which exhibited characteristic absorption peaks from 361 to 395 nm for the nanoparticles synthesized under microwave irradiation for 1 to 5 min. and was free of impurity peaks (Fig. 3). However, the red shift in absorption peaks (from 361 to 395 nm) of ZnO synthesized under microwave irradiation from 1 to 5 min. is due to the formation of bigger nanoparticles and / or formation of nanoaggregates. The formation of bigger nanoparticles and/or nanoaggregates with increase in microwave irradiation time can also be explained by the DLS experiments which exhibited hydrodynamic size from 135 to 361 nm for the nanoparticles synthesized under microwave irradiation for 1 to 5 min (Fig. 4).

### 3.2 Quercetin loading on ZnO surface and characterization

After confirming the synthesis of ZnO, quercetin was loaded onto ZnO surface via the metal ion–ligand coordination bond. Quercetin loading did not much affect the size of ZnO as evident from the small shift (3 nm) in λ_max_ and small increase (12 nm) in hydrodynamic size (Fig. 4). The attachment of quercetin on the surface of the nanoparticle was also confirmed by analyzing FTIR spectra. The spectrum of quercetin-loaded ZnO (ZnO/QR) showed multiple distinctive peaks of quercetin from 1000 cm^−1^ to 1700 cm^−1^ (Fig. 2) [41]. The broad and high intensity peak at 3410 cm^−1^ can be assigned to the stretching band of −OH groups. The peak at 2937 cm^−1^ is due to the stretching band of C—H groups; the peak at 1667 cm^−1^ is due to the stretching band of C=O groups; peaks at 1612, 1560, 1492 and 1412 cm^−1^ are due to the presence of aromatic ring; the peaks at 1361 and 1323 cm^−1^ are due to C—O—H bending, and the peaks at 1200–1000 and 1266 cm^−1^) are due to C—O stretching. These results indicate that quercetin was successfully loaded onto the ZnO.

**Fig. 5:**
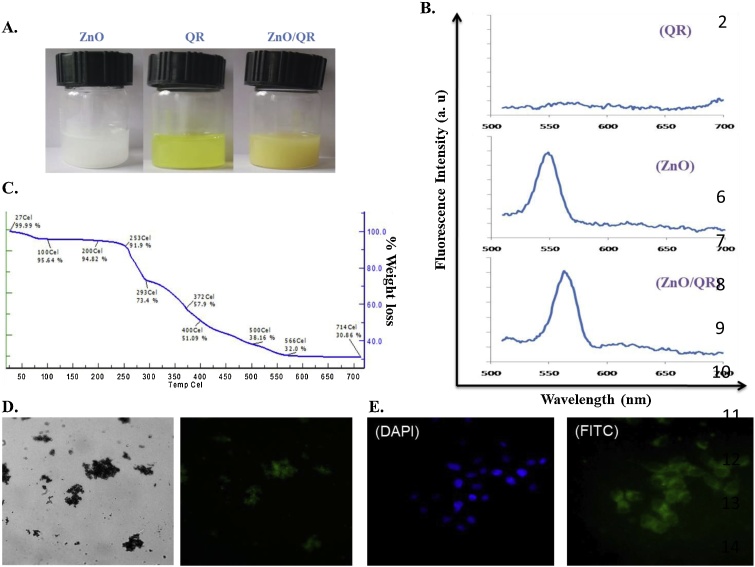
Optical/fluorescence images, fluorescence spectrum, TGA and cellular uptake of ZnO and/ or ZnO/QR nanorods: (A) Optical images of quercetin, ZnO4 (ZnO) and ZnO/QR nanorods respectively. (B) Fluorescence spectrum of quercetin, ZnO4 (ZnO) and ZnO/QR nanorods respectively. (C) TGA Analysis of ZnO/QR nanorods. (D) Bright field and green fluorescence images of ZnO4 (ZnO) nanorods. (E) Intracellular uptake of the ZnO/QR showing FITC fluorescence, fluorescent micrographs obtained at 40X magnification, DAPI fluorescence indicates the nucleus of the cells.

The process of quercetin conjugation on the ZnO surface was also monitored by optical images (Fig. 5A) and fluorescence spectral analysis (Fig. 5B). Initial ZnO were white in color but after quercetin absorption the color was changed to yellowish brown (Fig. 5A). ZnO exhibit a strong emission peak at 550 nm, while the loaded sample emits at a longer wavelength of 563 nm, indicating interaction with quercetin. This observed red shift may be due to the fact that energy is lost not because of fluorescent emission of photons but because of other forms such as vibration [42]. Further, TGA estimated the amount of quercetin loaded on ZnO surface (Fig. 5C). The weight loss graphs show few-step profiles for ZnO/QR. The melting point of pure quercetin is around 316 °C. Therefore, the first 27–200 °C weight loss corresponded to the evaporation of water and adsorbed low weight solvents or reagents [43]. The second weight loss curve from 250 to 300 °C corresponded to the decomposition of residual Zn(OH)_2_. The weight loss steps between 300 and 700 °C indicated the absolute amount of quercetin attached on ZnO surface, i.e., 42% (Table 1).

**Table 1:** Drug loading content (DLC) and drug loading efficiency (DLE) of ZnO/QR nanorods.

Feed ratio, ZnO:QR (w/w)	DLC (%)	DLE (%)
1:1	42	72.4

### 3.3 Intra cellular uptake of ZnO/QR

Since the synthesized ZnO are showing strong green fluorescence when excited by UV light (Fig. 5D), therefore, we could expect that these nanorods can be a promising candidate for bioimaging applications. Biolabeling with ZnO via endocytosis in cancer cells was verified by fluorescence microscopy. MCF-7 cells were incubated with ZnO for 6 h. The definite green fluorescence in thecytoplasmic compartment indicated the endocytosis-mediated penetration of ZnO into the cells (Fig. 5E).

### 3.4 *In vitro* quercetin release from ZnO/QR quercetin release from ZnO/QR

**Fig. 6:**
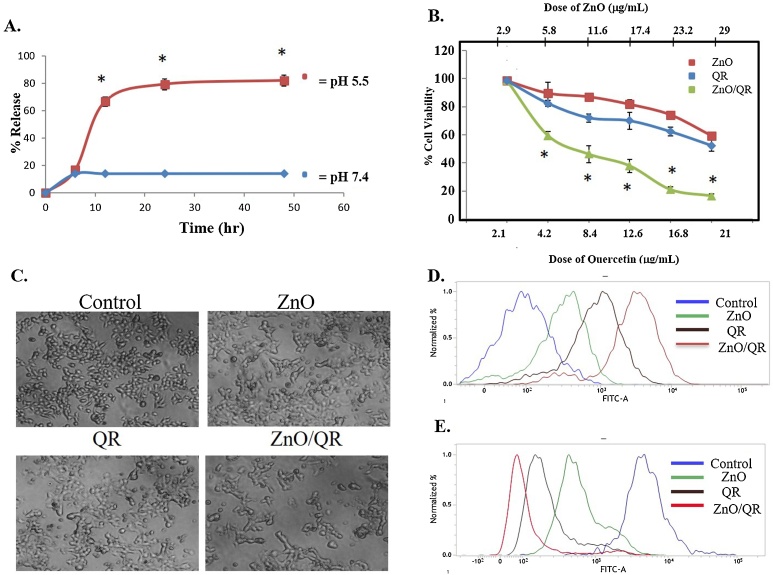
pH-dependent drug release and in vitro cytotoxicity: (A) Quercetin release patterns of the ZnO/QR nanorods in PBS at varied pH conditions at 37 °C. The equivalent drug concentration was 1 mg/ml. “*” indicates significant difference values compared to pH-7.4. (p* < 0.05). (B) Cell viability of MCF-7 cells exposed to ZnO, free quercetin, or ZnO/QR for 48 h. All values are relative tothe control and expressed as mean ± SD. “*” indicates significant difference values compared to cells treated with only quercetin and only ZnO nanorods. (p* < 0.05). (C) Bright field micrographs obtained at 10X magnification. (D) Determination of intracellular ROS level using DCFDA staining by FACS analysis. (E) Determination of mitochondrial membrane potential using Rhodamine 123 staining by FACS analysis.

The *in vitro* release of quercetin from ZnO/QR was quantified in a time-dependent manner at normal physiological conditions (pH 7.4) and in mild acidic conditions (pH 5.0) mimicking the endo-lysosomal pH. At pH 7.4 quercetin was released very slowly from ZnO/QR and finally, release of quercetin was only about 14% after 48 h (Fig. 6A). At acidic conditions, the release of quercetin from ZnO/QR was found to be accelerated. The cumulative release of quercetin from ZnO/QR could reach as high as about 82% within 48 h, which was approximately 6 times more than that observed at pH equivalent to physiological level (Fig. 6A). This result showed that the release of quercetin from ZnO/QR was pH-responsive. The pH dependent release of QR at pH 5.5 can be attributed towards the partial dissolution property of ZnO at mild acidic conditions [26]. Together with this the complexing ability of the catechol-type B ring of quercetin with metal ion requires both 3′ and 4′—OH groups to be dissociated [44]. However, at low pH, the stability of the complex is less because the flavonoids are predominantly present in their undissociated form, leading to breaking of metal-ligand bonds. Therefore, the release of drug would be minimum at physiological condition and maximum release would be observed at cancer cells.

### 3.5 Anti-cancer activity of ZnO and ZnO/QR

The *in vitro* cytotoxicity of quercetin, ZnO nanorods and quercetin-loaded ZnO (ZnO/QR) nanorods was evaluated using a 3-(4,5-dimethylthiazol-2-yl)-2,5-diphenyltetrazolium bromide (MTT) assay. Human breast cancer cell line, MCF-7 was used for this assay. The cells were exposed to different concentrations of quercetin and nanoformulations for 48 h. The cytotoxic potential was evaluated thereafter. As depicted in Fig. 3B, quercetin, ZnO and ZnO/QR exhibited dose-dependent cytotoxicity in MCF-7cells. Among the different experimental groups, ZnO/QR were found to induce a higher cytotoxicity compared to free quercetin and ZnO nanorods for all test concentrations (Fig. 6B). Quercetin appeared to have less cytotoxicity than ZnO/QR because of its poor *in vitro* bioavailability without a carrier to deliver on the target cells [45]. A pH induced regulated release of quercetin from the ZnO/QR in cancer cells made ZnO/QR as a potential drug delivery agent for quercetin. Besides, a synergistic effect has also been simultaneously operating by partial dissolution of ZnO nanorods at acidic pH in cancer cells that contributed higher reactive oxygen species (ROS) formation together with the anticancer effects of quercetin resulting in greater efficacy of ZnO/QR. The cytotoxic potential was further confirmed by analysing the phase contrast images of the ZnO, quercetin and ZnO/QR exposed cells. The cytotoxic lesion of ZnO/QR was found to be higher compared to ZnO and free QR exposed cells (Fig. 6C).

To investigate the underlying principle behind the cytotoxicity of ZnO/QR, intracellular ROS level and MMP was determined [[46], [47], [48], [49], [50], [51]]. In accordance to the cell viability results, it was observed that ZnO/QR could significantly enhance the level of intracellular ROS (Increasing green fluorescence) and decreases the MMP (decreasing green fluorescence), thereby increasing mitochondrial dysfunction and caused cell death (Fig. 6D, E). Disruption of redox homeostasis is considered as an effective strategy in selectively killing the cancer cells without causing toxicity in the normal cells [52]. A combinatorial effect of ZnO/QR was observed compared to either ZnO or QR for ROS, MMP and cytotoxicity.

### 3.6 Antibacterial activity of ZnO/QR

**Fig. 7:**
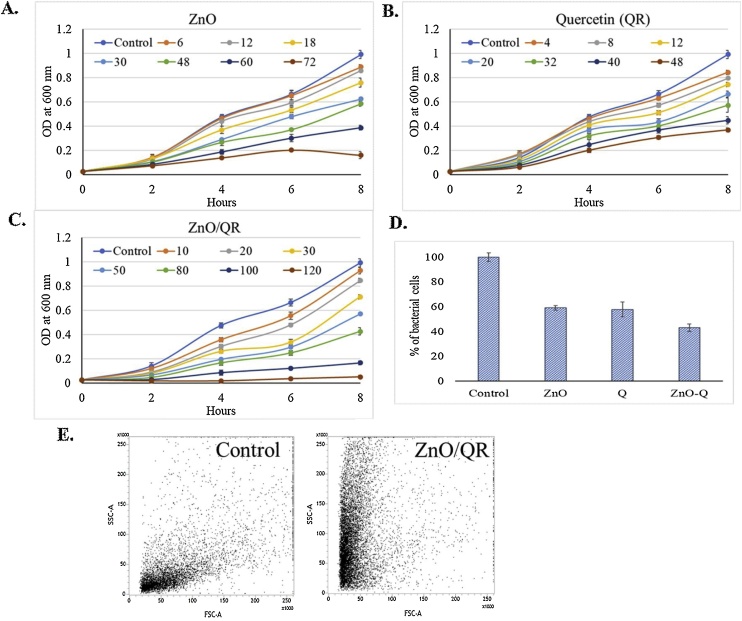
Antibacterial activity of ZnO/QR nanohybrids: (A–C) Growth curves of *E. coli* culture exposed to different concentrations of synthesized NPs. (D) *E. coli* are exposed to 80 μg/ml ZnO/QR. (E) FACS analysis of ZnO/QR adherence on *E. coli* cells.

Growth kinetic studies with the *E. coli* cells in presence of ZnO/QR nanocomplex indicates that the synthesized nanorods showed dose and time dependent growth inhibitory activity. Moreover, the results also showed that the growth inhibitory activity of ZnO/QR is higher than equivalent amount ZnO or quercetin (Fig. 7A, B, C). It can be concluded that quercetin can effectively sensitizes the bacterial cells to ZnO. It was determined that at 80 μg/mL ZnO/QR nanocomplex can inhibit bacterial growth by 57%, whereas, 41–43% growth inhibition was observed when the bacterial cells were exposed to only ZnO (48 μg/ml) or quercetin (32 μg/ml) (Fig. 7D).

### 3.7 Interaction of ZnO/QR and bacterial cells

FACS study indicated that the exposure to ZnO/QR nanocomplexes at a dose of 80 μg/mL for 8 h increase the cell granularity and decreases the size of the cells (Fig. 7E). It can be concluded from this result that absorption of the nanorods to the bacterial membrane causes the increase in SSC signal and the cytotoxicity caused by the nanorods results into the decrease of FSC signal [53].

## 4 Conclusions

Overall, the results indicate the effectiveness of the synthesized ZnO nanorods as a potential candidate for bioimaging and drug delivery purpose. In this study, it was found that ZnO/QR can induce profound cytotoxic effect via the combinatorial ROS enhancement effects of ZnO and free QR in cancer cells. Apart from its anticancer effect, ZnO and ZnO/QR showed profound antibacterial activity. Overall this study leads to the development of a multifunctional nanomaterial with enormous prospective for application in biomedicine.

## 5 Competing financial interests

The authors declare no competing financial interests.

## Conflict of interest

The authors have declared no conflict of interest.
